# Location‐specific cutaneous electrical stimulation of the footsole modulates corticospinal excitability to the plantarflexors and dorsiflexors during standing

**DOI:** 10.14814/phy2.15240

**Published:** 2022-07-01

**Authors:** Gagan Gill, Davis A. Forman, Joanna E. Reeves, Janet L. Taylor, Leah R. Bent

**Affiliations:** ^1^ Department of Human Health and Nutritional Sciences University of Guelph Guelph Ontario Canada; ^2^ Department for Health University of Bath Bath United Kingdom; ^3^ School of Medical and Health Sciences Edith Cowan University Perth Western Australia Australia; ^4^ Neuroscience Research Australia Randwick New South Wales Australia

**Keywords:** cervicomedullary junction stimulation, cutaneous reflexes, plantar sole, transcranial magnetic stimulation, transcortical pathway, cutaneous electrical stimulation

## Abstract

Non‐noxious electrical stimulation to distinct locations of the foot sole evokes location‐specific cutaneous reflex responses in lower limb muscles. These reflexes occur at latencies that may enable them to be mediated via a transcortical pathway. Corticospinal excitability to the plantarflexors and dorsiflexors was measured in 16 participants using motor evoked potentials (MEPs). Spinal excitability was measured in eight of the original participants using cervicomedullary motor evoked potentials (CMEPs). Measurements were collected with and without preceding cutaneous stimulus to either the heel (HEEL) or metatarsal (MET) locations of the foot sole, and evoked potentials were elicited to coincide with the arrival of the cutaneous volley at either the motor cortex or spinal cord. Plantarflexor MEPs and CMEPs were facilitated with cutaneous stimulation to the HEEL for MEPs (soleus *p* = 0.04, medial gastrocnemius (MG) *p* = 0.017) and CMEPs (soleus *p* = 0.047 and MG *p* = 0.015), but they were unchanged following MET stimulation for MEPs or CMEPs. Dorsiflexor MEPs were unchanged with cutaneous stimulation at either location, but dorsiflexor CMEPs increased with cutaneous stimulation (*p* = 0.05). In general, the increase in CMEP amplitudes was larger than the increase in MEP amplitudes, indicating that an increase in spinal excitability likely explains most of the increase in corticospinal excitability. The larger change observed in the CMEP also indicates that excitability from supraspinal sources likely decreased, which could be due to a net change in the excitability of intracortical circuits. This study provides evidence that cutaneous reflexes from foot sole skin are likely influenced by a transcortical pathway.

## Graphical abstract

Stimulation of foot sole skin has been shown to contribute significantly to modulation of locomotor muscle activity. Our investigation sought to uncover whether cutaneous stimulation to the heel and metatarsal regions of the foot sole influence measures of corticospinal and spinal excitability, in order to shed light on transcortical control of foot placement during gait. Our results demonstrated that both corticospinal and spinal excitability were elevated following cutaneous stimulation, although this effect differed based on stimulus site and muscle (plantarflexors or dorsiflexors). The findings of this study provide further evidence that transcortical pathways are likely involved in the cutaneous reflex, and they have the potential to drive changes in neurorehabilitative guidelines.

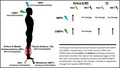

## 1 INTRODUCTION

Cutaneous afferent feedback from the soles of the feet is important for the control of balance (Kavounoudias et al., 2001; Oddsson et al., 2004). Previous work has shown that a reduction in plantar skin feedback can impair standing balance (McKeon & Hertel, 2007; Nurse & Nigg, 2001; Perry et al., 2000) and alter gait parameters (Eils et al., 2004), while enhancement of cutaneous afferent feedback from the feet has been shown to improve postural control (Galica et al., 2009; Perry et al., 2008; Priplata et al., 2003). These postural responses may be mediated through cutaneous reflexes, such as those highlighted by Fallon and colleagues (2005) where activation of cutaneous afferents from the foot sole reflexively modulates muscle activity in the lower limb. This study largely proposes reflex activation within spinal circuitry; however, transcortical pathways may also be involved.

When elicited through non‐noxious electrical stimulation, cutaneous reflexes commonly exhibit an initial increase in muscle activity (E1: thought to be mediated by an oligosynaptic spinal pathway), followed by a short period of inhibition (I1: also mediated at the spinal level, but dependent on descending signals), and then a second excitatory response (E2) (Issler & Stephens, 1983; Jenner & Stephens, 1982). With an approximate latency range of 70–110 ms in the lower limb, this later phase of the cutaneous reflex was proposed by Nielsen and colleagues (1997) to be at least partially transcortical in origin. In their study, subthreshold transcranial magnetic stimulation (TMS), paired with cutaneous stimulation of peripheral nerves at the ankle, increased tibialis anterior (TA) motor unit discharge rates and facilitated H‐reflex amplitudes. Importantly, these facilitatory effects were not present with electrical stimulation of the motor cortex, suggesting that the reflex pathway likely involved cortical circuits (Nielsen et al., 1997). This previous work focused on foot dorsum for purposes of dynamic stumbling corrective responses from the activation of dorsal skin. The foot sole skin is differentially engaged during different phases of the gait cycle, and is known to modulate activity of the lower limb musculature (Zehr & Stein, 1999). The significance of a possible transcortical connection from the plantar sole to the lower limb is that it would support higher level integration in balance and propulsive locomotor activities.

Reflex work in the lower limb suggests that the influence of cutaneous afferent stimulation on corticospinal excitability is likely more complex than widespread inhibition. Nakajima et al. (2006) demonstrated that plantarflexor (both soleus and medial gastrocnemius) muscle activity was facilitated following cutaneous stimulation of the heel but inhibited following forefoot (metatarsal) stimulation. In contrast, the tibialis anterior was facilitated and inhibited by forefoot and heel stimulation, respectively. This location specificity has also been observed in studies utilizing the stretch and H‐reflex; spinal excitability to the soleus is facilitated with cutaneous stimulation of the heel (Sayenko et al., 2007, 2009) but inhibited with stimulation of the metatarsals (Knikou, 2007; Sayenko et al., 2009). It is possible that cutaneous stimulation of the foot sole could also modulate corticospinal excitability to lower limb muscles in a similar location‐dependent manner; however, no such studies have been performed to date. If so, this would add important knowledge to the current understanding of how cutaneous activity influences locomotor outputs; cutaneous activity has been shown to aid in the production of sensory steering during walking, but this effect is thought to occur via reflexes at the spinal cord level (Zehr et al., 2014). Should cutaneous reflexes, elicited by stimulation at the foot sole, include a transcortical component, this would suggest cutaneous activity has a more sophisticated and complex involvement in the control and steering of locomotor outputs.

The purpose of the present study was therefore to examine corticospinal and spinal excitability to the plantarflexors and dorsiflexors following cutaneous afferent stimulation at either the heel or metatarsal locations of the foot sole. Our hypotheses were twofold: (1) cutaneous afferent stimulation would modulate corticospinal and spinal excitability in a similar location‐dependent manner as has previously been demonstrated in EMG and stretch/H‐reflex studies; and (2) the relative changes in corticospinal and spinal excitability following cutaneous afferent stimulation would not be equal, suggesting that supraspinal mechanisms contribute at least partially to the changes in corticospinal excitability.

## 2 METHODS

### 2.1 Subjects

Sixteen healthy subjects (8 female) aged 19–29 years (23 ± 3 years, mean ± SD) participated in this study. Subjects completed health history questionnaires and gave written informed consent prior to data collection. Subjects were free from neurological and musculoskeletal disorders. Subjects were excluded if they had a history of seizures, concussions, or lower limb injuries. All experimental procedures conformed to the standards set by the Declaration of Helsinki and were approved by the research ethics board at the University of Guelph (REB#16‐12‐520).

### 2.2 Cutaneous stimulation

**FIGURE 1:**
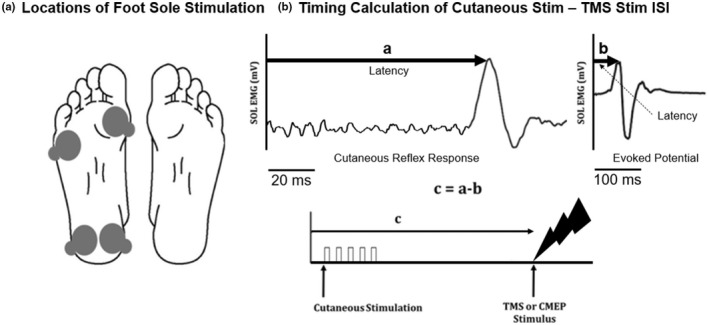
(A) Locations of plantar sole stimulation over the metatarsal and heel regions. Circles represent placement of electrodes on the right foot sole for stimulation of either the metatarsal or heel locations. (B) Timing of cutaneous stimulation relative to cortical and cervicomedullary stimulation. **a** latency (ms) is the time to the peak reflex response (occurring between 70 and 110 ms) measured in the soleus (SOL) following 100 electrical stimulations to either the HEEL or MET skin locations on the foot sole. **b** latency (ms) is the time to the peak of the evoked potential in the SOL occurring with TMS or cervicomedullary stimulation (CMEP). This is the estimated efferent conduction time. The **c** (ms) value, which is the interstimulus interval between the train of electrical stimulation to the plantar sole and the TMS or CMEP pulse, is calculated by **a − b**. Thus, **c** is the estimated afferent conduction time from the foot sole to the motor cortex. **c**was calculated separately for TMS and CMS as these techniques have different latencies.

To evoke the cutaneous reflex responses, the heel and metatarsal locations of the plantar foot sole were electrically stimulated using a constant‐current stimulator (Model DS7AH, Digitimer) and two pairs of Ag/AgCl stimulus electrodes (1 inch diameter) placed on both the metatarsal and heel locations of the right plantar sole (Figure 1A). The metal tabs of the electrodes were left exposed on the lateral borders of the plantar sole locations to allow the stimulator leads to be connected, and the anode and cathode arrangement varied between participants to obtain local skin sensation across the target foot region (determined based on subject feedback). Electrode gel was applied on the electrodes to reduce impedance and lower the voltage necessary to maintain a constant current. Electrical stimulation consisted of a train of five constant‐current rectangular pulses (each 1.0 ms duration, inter‐pulse interval 3 ms), with trains applied at a frequency of 200 Hz (Nakajima et al., 2006).

Perceptual threshold (PT), defined as the lowest stimulus intensity (current) that evoked detectable tactile sensations at the cutaneous site, was determined for each location. PT was measured with the subjects standing while the experimenter gradually decreased the stimulus voltage until the participant could barely discern the stimulus (identified as PT) on their plantar sole. Electrical stimulation intensity was set at 2 times the perceptual threshold (2.0 × PT) during testing to evoke a non‐noxious cutaneous sensation during each trial. It was verbally confirmed by all subjects that the stimulation intensity was not painful.

### 2.3 Transcranial magnetic stimulation

Transcranial magnetic stimulation (TMS) was used to investigate the influence of foot sole stimulation on corticospinal excitability. TMS was performed using two MagStim 200 stimulators connected to a Bistim module and a double cone coil (MagStim). The coil was placed over the right leg area of the motor cortex (slightly lateral to the vertex). To locate the stimulation site, participants were seated with their legs relaxed and knees bent ~90° while stimuli were delivered through the coil. The stimulation site was identified as the location over which motor evoked potentials (MEP) to the soleus could be evoked with the lowest stimulation intensity. Once the optimal site was identified, subjects stood for the experimental procedures. The TMS intensity (% of maximal stimulator output [%MSO]) was then adjusted in the standing position to produce reliable and repeatable MEPs of at least 100 μV peak‐to‐peak amplitude. During the experimental protocol, TMS was delivered as described below. A single pulse was applied using the MagStim stimulator (a range of 40%–65% of maximal stimulator output was used across participants to elicit a 100 μV amplitude MEP in the soleus).

### 2.4 Cervicomedullary stimulation

The corticospinal tract was also stimulated noninvasively at the level of the cervicomedullary junction through transmastoid electrical stimulation (cervicomedullary stimulation, CMS) in 8 of the original 16 subjects. CMS was performed using a 200 μs electrical pulse (Model D7SAH, Digitimer) delivered through a pair of electrodes (Clear Trace, ConMed) fixed to the skin just posterior to the mastoid processes with the cathode on the left. Stimulation intensity was set at a level at which a cervicomedullary motor evoked potential (CMEP) could be observed in the soleus during standing. The stimulation intensity (200–300 mA) was adjusted to produce CMEPs to match the amplitude of the previously recorded unconditioned MEP amplitude of each participant (roughly100 μV).

### 2.5 Muscle recordings

Both MEPs and CMEPs were recorded using surface electromyography (EMG). EMG was recorded on the right leg from the soleus (SOL), medial gastrocnemius (MG), and tibialis anterior (TA) muscles using pairs of surface Ag/AgCl electrodes (Ambu Blue Sensor). Electrodes were placed in a monopolar arrangement with one electrode over the muscle belly and a second along the muscle‐tendon interface of each muscle; for SOL the proximal electrode was placed laterally along the soleus border, with the distal electrode on the Achilles tendon, for TA, the proximal electrode was placed distal and lateral to the tibial tuberosity, over the proximal muscle belly, with the distal electrode where the tendon crosses over the tibia, and finally the MG electrodes were placed on the medial head of the gastrocnemius, and the proximal Achilles tendon. EMG signals were amplified (gain 500–1000, band passed filtered between 10 to 1000 Hz; Bortec AMT‐8 system, Bortec Biomedical Ltd), and digitized at 2048Hz (Spike 2 version 7, Cambridge Electronic Design).

### 2.6 Study design

#### 2.6.1 Timing of cutaneous afferent stimulation relative to TMS and CMS

To coordinate the timing of TMS and CMS with the arrival of the stimulated cutaneous input, cutaneous reflex responses to foot sole electrical stimulation were evoked and the latency of the cutaneous reflex in soleus was calculated for each individual subject.

Cutaneous reflexes were evoked by stimulation of the metatarsal or heel locations of the right plantar sole while subjects stood upright on a stable surface with their eyes open. In order to obtain stable and clear cutaneous reflexes, 100 stimulations were applied at each location (heel or metatarsal), with a 3‐min resting period between locations. The amplified soleus EMG signal was digitally rectified, and averaged to the stimulus onset to calculate latency. A reflex response was considered present if it rose above or fell below three standard deviations of the mean background EMG for at least 8 ms (Nakajima et al., 2006). The time of the reflex in the soleus muscle in response to the electrical stimulation of the plantar sole (occurring in the range of 70–110 ms) was calculated as the time of the first peak, whether inhibitory or excitatory. This measurement was performed for reflex responses occurring following stimulation to both heel and metatarsal locations.

To estimate efferent conduction time from the motor cortex to the muscle, the average latency (to the first peak) of 5 soleus MEPs was calculated during standing. For the cervicomedullary protocol, efferent conduction time from the brainstem to the muscle was calculated from the average latency (to the first peak) of 5 CMEPs in the soleus.

Interstimulus intervals (ISIs) between cutaneous stimulation of the plantar sole and TMS (or CMS; calculations performed separately as MEPs and CMEPs demonstrate unique latencies) were calculated by subtracting MEP (or CMEP) latency from the latency of the cutaneous reflex. If the cutaneous reflex is transcortical, this timing coincides delivery of TMS with any motor cortical response to the cutaneous input. Whatever the pathway of the cutaneous reflex, the timing coincides the arrival of descending volleys elicited by TMS or CMS with the response of the motoneurons to the cutaneous input (Figure 1B).

#### 2.6.2 Experiment 1: Effect of foot sole stimulation on corticospinal excitability

In 16 subjects, we investigated the effect of foot sole cutaneous afferent stimulation on ankle plantarflexor and dorsiflexor MEPs during quiet stance. Data were collected while subjects stood with a natural base of support and eyes open. Participants received block randomized plantar stimulation at either the heel (HEEL) or metatarsal locations (MET) with an independent set of control trials completed for each location. In total 40 MEPs were elicited at each location: 20 control MEPs with no foot sole stimulation, and 20 MEPs with cutaneous electrical stimulation paired with TMS. The timing of the cutaneous—TMS ISI was based on the latencies of responses in soleus and was calculated for each subject (range 48–60 ms; see above). For all trials, the time between consecutive TMS pulses was 10 s. A 5‐min seated resting period was imposed between locations (HEEL or MET) to avoid a confounding effect arising from fatigue.

#### 2.6.3 Experiment 2: Effect of foot sole stimulation on spinal excitability

We also examined the effect of foot sole stimulation on evoked responses from direct activation of the corticospinal tract in 8 of the original 16 subjects (in separate experimental sessions). This was to establish if the observed MEP changes were supraspinal in origin. Data were again collected while subjects remained standing with their eyes open and maintained a natural base of support. Stimulations were elicited in the same two locations as the TMS session; HEEL stim and MET stim (randomized). Each location consisted of 15 randomized trials: 5 control CMEPs without cutaneous stimulation and 10 CMEPs paired with cutaneous stimulation. Interstimulus intervals were calculated specifically for each participant (range 57–68 ms).

### 2.7 Data analysis and statistics

The size of the MEPs and CMEPs were measured as the peak‐to‐peak amplitudes of the non‐rectified responses. Evoked potentials were analyzed in Signal Software version 6 (Cambridge Electronic Design; both MEP and CMEP). For CMEP amplitudes only, the 5 control CMEPs for the Heel trials and the 5 control CMEPs for the MET trials were averaged into a single control value. Background RMS EMG activity was measured from the EMG over a 50‐ms period before the stimulus to ensure there was a comparable level of EMG activity across conditions for each muscle. Statistical analysis was performed on the raw MEP and CMEP responses and those expressed as a percentage of difference from the NO FOOT STIM condition.

For the first hypothesis, two‐way repeated measures ANOVAs for each muscle (SOL, MG and TA) and for each potential (MEPs and CMEPs) assessed differences in evoked potential amplitudes that resulted from electrical stimulation of the plantar sole during standing. The two within‐subject factors (independent variables) were stimulation condition (NO FOOT STIM and FOOT STIM) and location (HEEL or MET). Peak‐to‐peak amplitudes for the raw MEPs (*n* = 16) and CMEPs (*n* = 8) of each muscle were analysed.

For the second hypothesis, MEPs and CMEPs (*n* = 8) responses, expressed as a percent difference from the NO FOOT STIM condition, were compared in a three‐way repeated measures ANOVA with independent variables of potential (MEP vs. CMEP), location (HEEL or MET), and muscle (Soleus, MG, and TA). Additionally, an a priori comparison was run to compare percent differences between MEP and CMEP within the HEEL location and within the MET location.

For all ANOVAs, normality and sphericity of response amplitudes were evaluated using Shapiro–Wilk and Mauchley's tests, respectively. If a violation of normality occurred, the data were log transformed prior to conducting the statistical analyses. Significance level was set as *p* < 0.05 for all analyses. Estimated effect sizes were calculated as partial eta squared (ηp^2^). Bonferroni post hoc analysis was applied for significant effects. All statistical analyses were performed using SPSS version 25 (IBM ).

## 3 RESULTS

### 3.1 Background EMG

The level of background EMG activity was quantified for all muscles (SOL, MG, and TA) during the 50‐ms period prior to the cutaneous stimulation onset. Background EMG was assessed to ensure there was a comparable level of EMG activity across stimulation conditions (NO FOOT STIM and FOOT STIM). There were no statistical differences in average background EMG for *experiment 1* between NO FOOT STIM and FOOT STIM for the SOL (Heel: *p* = 0.129, MET: *p* = 0.316), MG (Heel: *p* = 0.109, MET: *p* = 0.129), or TA (Heel: *p* = 0.981, MET: *p* = 0.665), nor were there any differences in background EMG for *experiment 2* between NO FOOT STIM and FOOT STIM for the SOL (Heel: *p* = 0.728, MET: *p* = 0.212), MG (Heel: *p* = 0.708, MET: *p* = 0.298), or TA (Heel: *p* = 0.467, MET: *p* = 0.829).

### 3.2 Experiment 1: Effect of foot sole stimulation on corticospinal excitability

**FIGURE 2:**
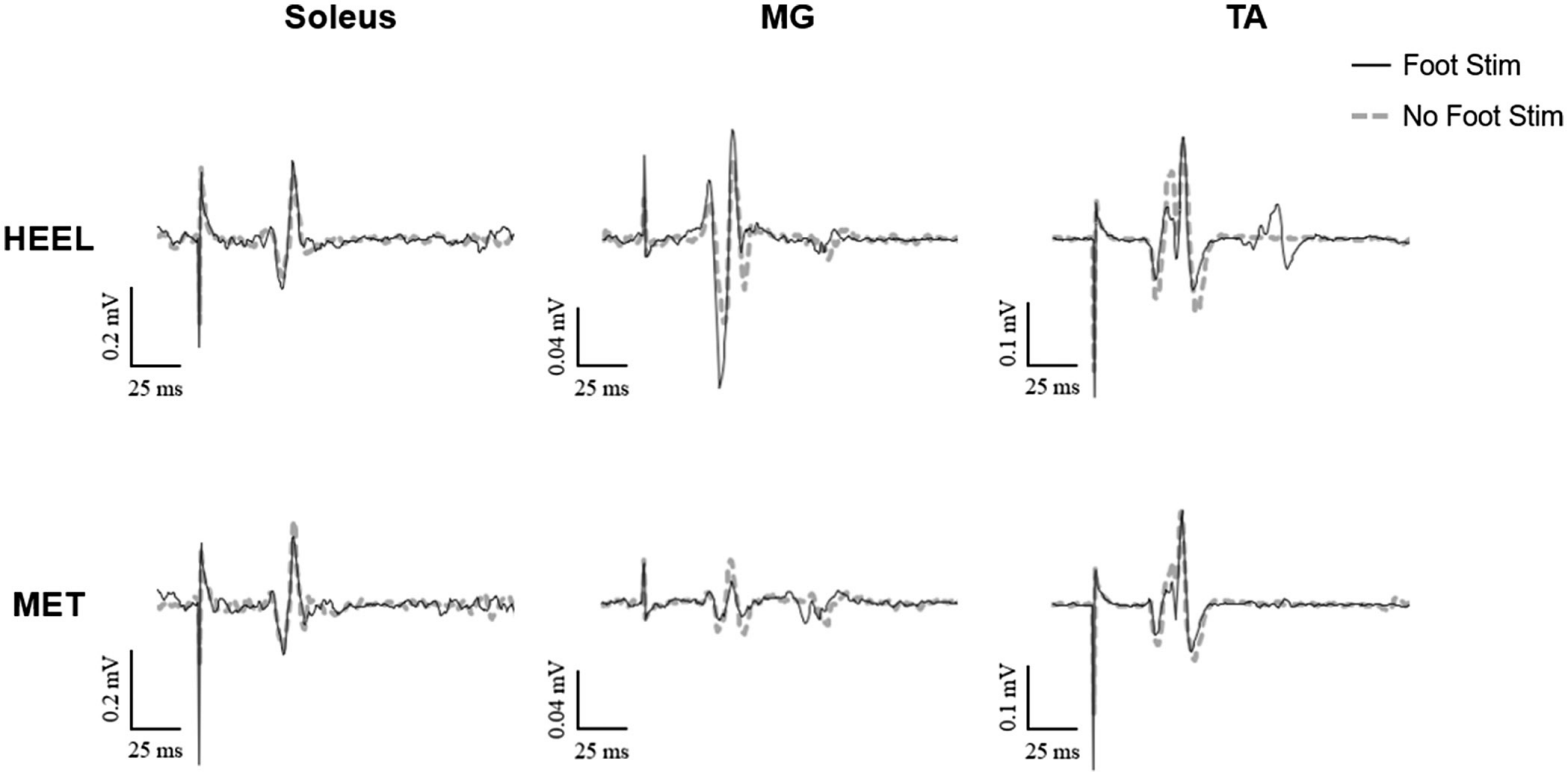
Average motor evoked potentials (MEPs) recorded from the soleus (SOL), medial gastrocnemius (MG) and the tibialis anterior (TA) of a representative individual during standing. Each trace is the average of 20 potentials. Solid black lines depict MEPs that were elicited following cutaneous foot sole stimulation; dashed gray lines depict MEPs that were elicited without preceding stimulation.

**FIGURE 3:**
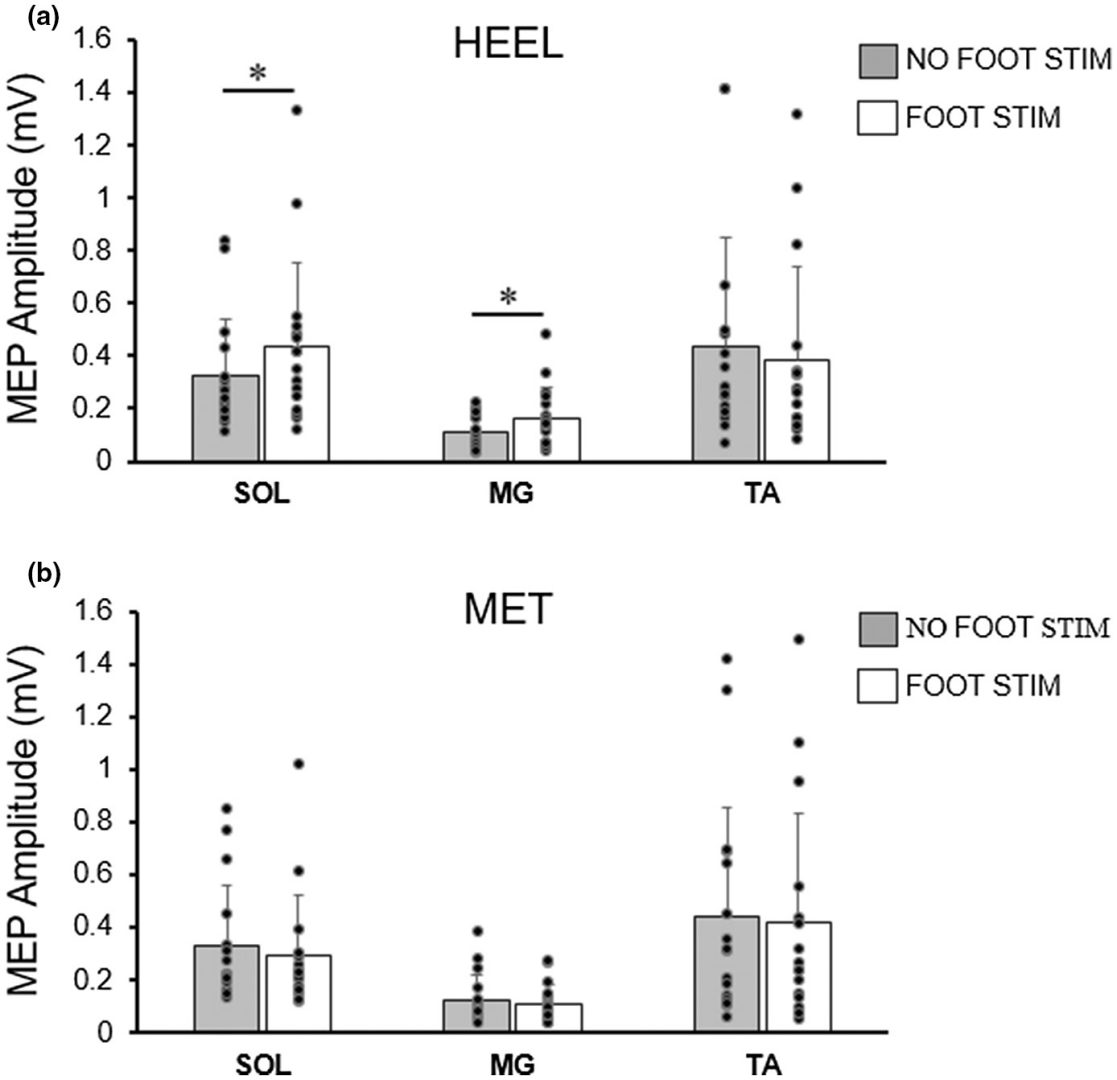
Group averages (means ± SD, *n* = 16) of MEP amplitudes elicited in the soleus (SOL), medial gastrocnemius (MG), and tibialis anterior (TA) with (white bars) or without (gray bars) preceding cutaneous foot sole stimulation. Individual data are represented by black circles. Foot sole stimulation was delivered at the heel (a) and the metatarsal (MET) locations (b). * indicates a significant difference between no foot stim and foot stim (*p < 0.05).

MEPs from all three muscles from a representative individual are shown in Figure 2. For this individual, SOL and MG MEPs were larger when preceded by cutaneous stimulation at the heel location. In all other conditions, stimulation resulted in decreased MEP amplitudes. For group data, MEPs were non‐normally distributed, so were log transformed. Electrical stimulation of the foot sole modulated the peak‐to‐peak amplitude of MEPs of the plantarflexor muscles (Figure 3). SOL MEPs showed a significant interaction for location by stimulation condition (*F*_(1,15)_ = 17.383, *p* = 0.001, ηp^2^ = 0.537). Changes in excitability differed with stimulation at the two locations. Post‐hoc comparisons determined that SOL MEP amplitudes significantly increased from NO FOOT STIM (0.32 ±0.22 mV) to FOOT STIM (0.43 ± 0.32 mV) at the HEEL location (Figure 3a; *p* = 0.04), but MEP amplitudes were unchanged from NO FOOT STIM (0.33 ± 0.23 mV) to FOOT STIM (0.29 ± 0.23 mV) at the MET location (Figure 3b; *p* = 0.077).

A significant interaction between location and stimulation condition was also found for MEP amplitudes for the MG (*F*_(1,15)_ = *p* = 0.002, ηp^2^ = 0.494). Similar to the soleus, post‐hoc comparisons revealed a significant increase in amplitude with stimulation at the HEEL location (Figure 3a; NO FOOT STIM: 0.11 ± 0.06 mV, FOOT STIM: 0.17± 0.12 mV; *p* = 0.017), but there was no significant change in MEP amplitudes when stimulus was applied to the MET location (Figure 3b; NO FOOT STIM: 0.12 ± 0.10 mV, FOOT STIM: 0.11 ± 0.08 mV; *p* = 0.053).

Electrical stimulation of the foot sole did not significantly modify the size of the MEPs of the TA. There was no significant interaction for location by stimulation condition (*F*_(1,15)_ = 0.169, *p* = 0.687, ηp^2^ = 0.011). While there was a slight decrease from NO FOOT STIM to FOOT STIM at both HEEL (0.44 ± 0.41 mV to 0.38 ± 0.36 mV) and MET (0.44 ± 0.41 mV to 0.42 ± 0.42 mV) locations (Figure 3a,b), there were no significant main effects for location (*F*_(1,15)_ = 0.130, *p* = 0.724, ηp^2^ = 0.009) or stimulation condition (*F*_(1,15)_ = 1.708, *p* = 0.211, ηp^2^ = 0.102).

### 3.3 Experiment 2: Effect of foot sole stimulation on spinal excitability

**FIGURE 4:**
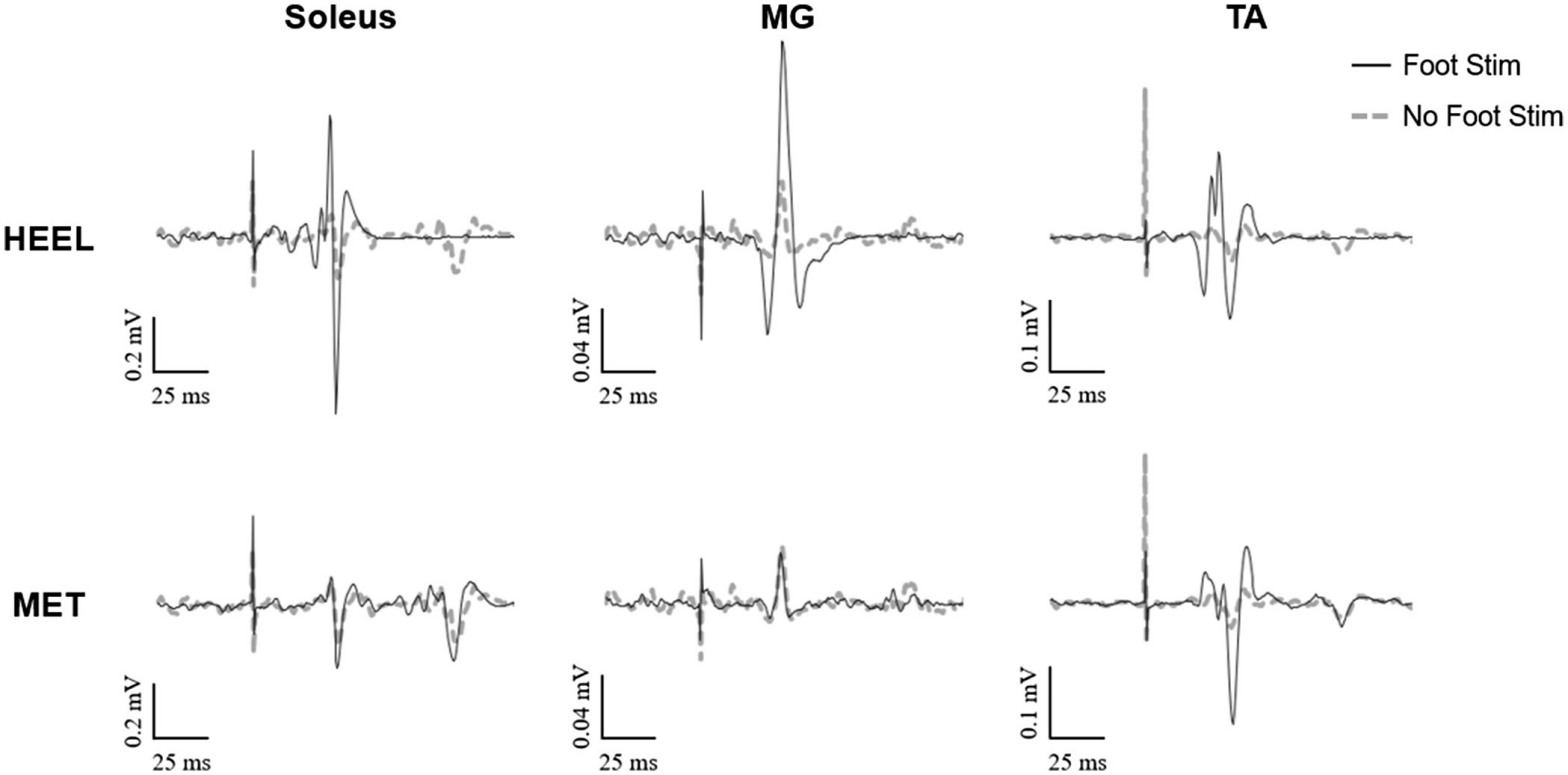
Average cervicomedullary motor evoked potentials (CMEPs) of the soleus (SOL), medial gastrocnemius (MG) and the tibialis anterior (TA) of a representative individual during standing. Each trace is an average of 10 potentials. Solid black lines depict CMEPs that occurred following cutaneous foot sole stimulation; dashed gray lines depict CMEPs that occurred without preceding stimulation.

**FIGURE 5:**
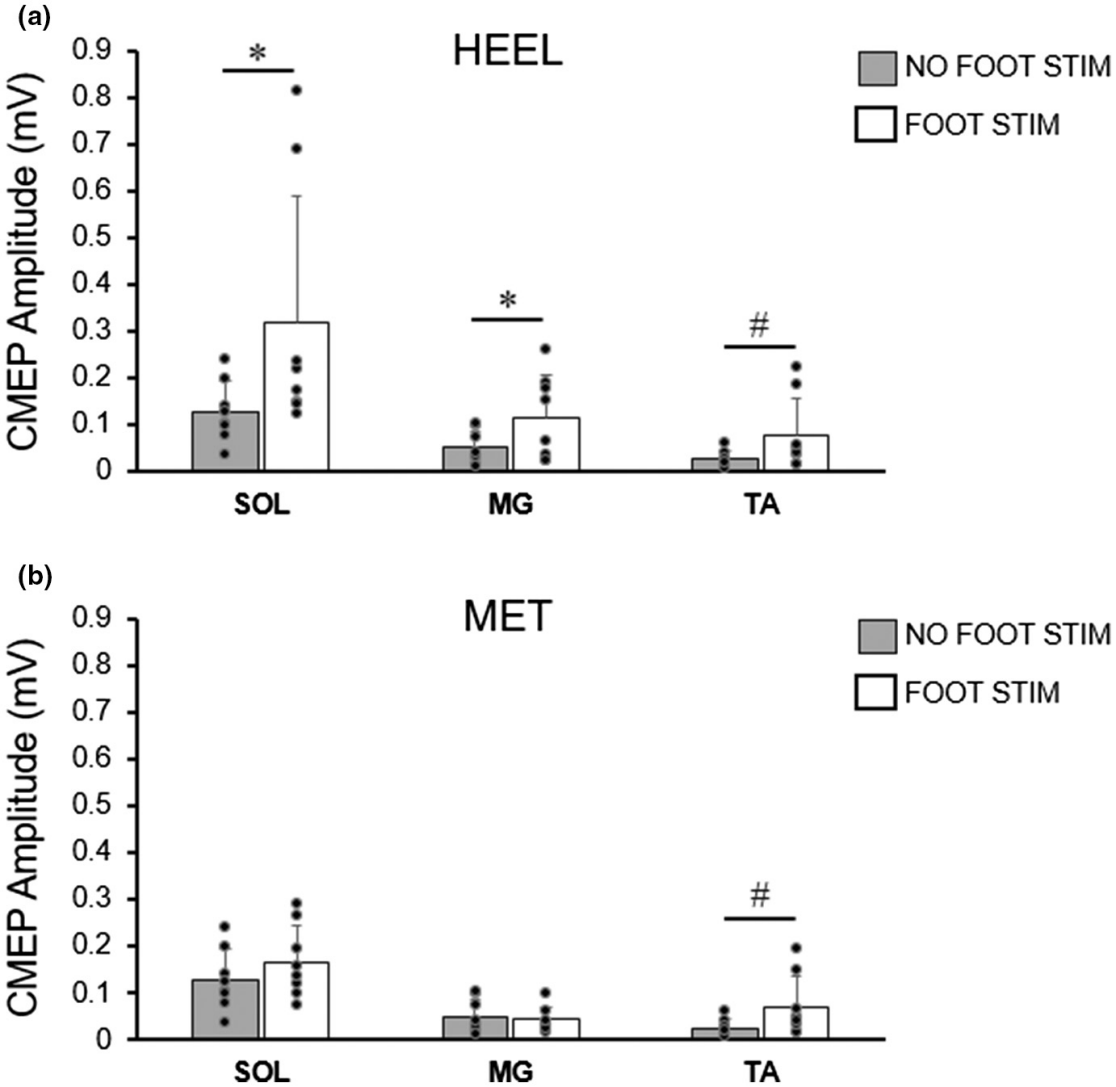
Group averages (means ± SD, *n* = 8) of CMEP amplitudes elicited in the soleus (SOL), medial gastrocnemius (MG), and tibialis anterior (TA) with (white bars) or without (gray bars) preceding sucutaneous foot sole stimulation. Individual data are represented by black circles. Foot sole stimulation was delivered at the heel (a) and the metatarsal (MET) locations (b). * indicates a significant difference between no foot stim and foot stim (*p < 0.05). # indicates a significant main effect across both stimulation locations (*p* < 0.05)

CMEPs from all three muscles from a representative individual are shown in Figure 4. For this individual, CMEPs were larger in all muscles following cutaneous stimulation at the heel location; CMEPs were only larger in the soleus and TA for stimulation at the MET location. For SOL group data (Figure 5), there was no interaction between location and stimulation condition on CMEP amplitudes; however there was a significant main effect of stimulation condition (*F*_(1,7)_ = 8.456, *p* = 0.023, ηp^2^ = 0.547). The *increase* in amplitude from NO STIM to FOOT STIM was significant for HEEL (Figure 5a; 0.13 ± 0.07 mV to 0.32 ± 0.27 mV, *p* = 0.047), but not for MET (Figure 5b; 0.13 ± 0.07 mV to 0.17 ± 0.08 mV, *p* = 0.146).

For MG CMEP amplitude, a significant interaction of location by stimulation condition was found (*F*_(1,7)_ = 7.567, *p* = 0.028, ηp^2^ = 0.519). Post hoc comparisons revealed a significant increase in CMEP amplitude with stimulation at the HEEL location (Figure 5a; NO FOOT STIM: 0.05 ± 0.04 mV, FOOT STIM: 0.12 ± 0.09 mV; *p* = 0.015), and no change at the MET location (Figure 5b; NO FOOT STIM: 0.05 ± 0.04 mV, FOOT STIM: 0.04 ± 0.03 mV; *p* = 0.506).

For TA CMEP amplitude, there was no significant interaction of location by stimulation condition; however a significant main effect of stimulation condition (*F*_(1,7)_= 5.616, *p* = 0.05, ηp^2^ = 0.445) was found, where CMEP amplitude increased following cutaneous stimulation. Although FOOT STIM significantly increased the CMEP when grouped, post‐hoc comparisons revealed no difference in CMEP amplitudes between stimulus conditions at either the HEEL (0.03 ± 0.02 mV to 0.08 ± 0.08 mV, *p* = 0.059) or the MET locations (0.03 ± 0.02 mV to 0.07 ± 0.07 mV, *p* = 0.052) although they approached statistical significance.

### 3.4 Effect of foot sole stimulation on MEPs versus CMEPs

To determine whether the changes in corticospinal excitability following foot sole stimulation were driven by changes at the supraspinal or spinal level, we compared the percent change (relative to NO STIM trials) in conditioned MEPs and CMEPs in the eight subjects who participated in both protocols. We first used paired *t*‐tests to examine whether there were differences between the average MEP NO FOOT STIM amplitude and the CMEP NO FOOT STIM responses for each muscle. MEPs were significantly larger than CMEPs for the SOL (Heel: *p* = 0.03, MET: *p* = 0.022) and the TA (Heel: *p* = 0.001, MET: *p* = 0.014). There were no significant differences for MG (Heel: *p* = 0.242, MET: *p* = 0.222).

**FIGURE 6:**
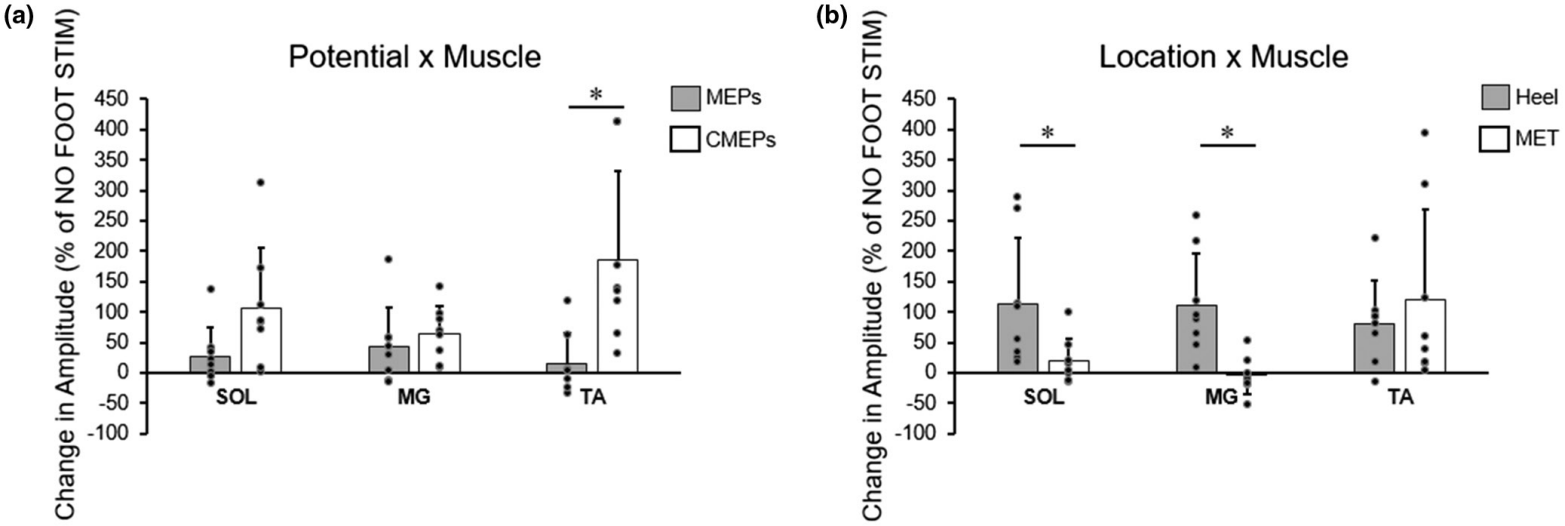
Group averages (means ± SD, *n* = 8) of MEP and CMEP amplitudes, expressed as a percent change from control (no foot stimulation) trials, elicited in the soleus (SOL), medial gastrocnemius (MG), and tibialis anterior (TA). Gray and white bars depict MEP amplitudes and CMEP amplitudes, respectively, in panel a. Gray and white bars depict cutaneous foot sole stimulation at either the heel or metatarsal (MET) location, respectively, in panel b. Individual data are represented by black circles. Results are collapsed across location (a) and potential (b) to depict findings from 3‐way repeated measures ANOVA. * indicates a significant difference between either potentials (MEPs vs. CMEPs) or between locations (HEEL vs. MET) within a given muscle (*p < 0.05)

For each subject, conditioned MEPs and CMEPs were expressed as the percent change from the matching NO FOOT STIM values (Figure 6). Although there was no three‐way interaction between muscle, location, and potential, there were two‐way interactions for both potential × muscle and muscle × location (see Table 1 for full three‐way ANOVA results). For the two‐way interaction for potential × muscle, post‐hoc paired *t*‐tests between potentials (MEPs vs. CMEPs) for each muscle showed that stimulation‐related increases in CMEP amplitudes were significantly larger than those in MEPs for the TA (186% ± 147% vs. 15% ± 50%, *p* = 0.003), but there were no differences between potentials for either SOL or MG. For the muscle × location interaction, post hoc paired *t*‐tests between locations (HEEL vs. MET) for each muscle showed that HEEL stimulation resulted in larger evoked potential increases than MET for the SOL (114% ± 108% vs. 19% ± 38%, *p* = 0.047) and MG (112% ± 85% vs. −4% ± 30%, *p* = 0.01), but not for the TA (Figure 6).

**TABLE 1:** Summary of results from the three‐way repeated measures ANOVA. Muscle (SOL, MG, TA), Location (HEEL, MET) and Potential (MEP, CMEP). Astrick indicates a significant p‐value equal to or less than 0.05.

	*F* statistic	*p*‐value	Effect size
Muscle	*F*_(2,14)_ = 2.240	0.143	0.242
Potential	*F*_(1,7)_ = 15.880	0.005^*^	0.694
Location	*F*_(1,7)_ = 3.003	0.127	0.300
Muscle × potential	*F*_(2,14)_ = 5.684	0.016^*^	0.448
Muscle × location	*F*_(2,14)_ = 8.141	0.005^*^	0.538
Potential × location	*F*_(1,7)_ = 0.063	0.809	0.009
Muscle × potential × location	*F*_(2,14)_ = 0.241	0.789	0.033

**FIGURE 7:**
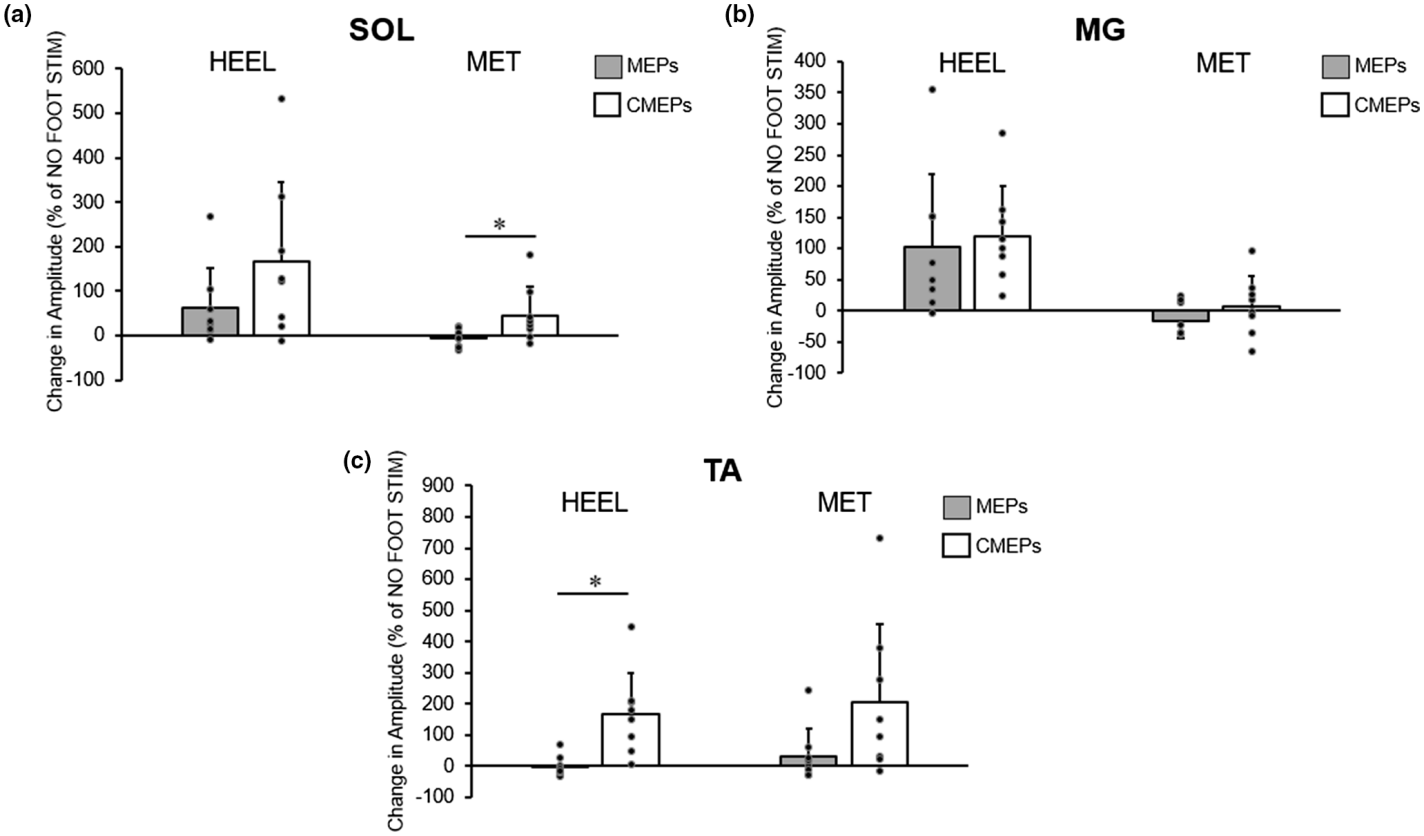
Group averages (means ± SD, *n* = 8) of MEP and CMEP amplitudes, expressed as a percent change from control (no foot stimulation) trials, elicited in (a) the soleus (SOL), (b) medial gastrocnemius (MG), and (c) tibialis anterior (TA). Gray and white bars depict MEP amplitudes (from experiment 1 data) and CMEP amplitudes (from experiment 2 data), respectively, following cutaneous foot sole stimulation at either the heel or metatarsal (MET) locations. Individual data are represented by black circles. * indicates a significant difference between potentials (MEPs vs. CMEPs) at a single location, determined via a priori planned paired *t*‐tests

Finally, a priori planned comparisons between the changes in the conditioned MEP and CMEP amplitudes (% of NO STIM) were performed. For SOL, the conditioned CMEP was significantly larger than the conditioned MEP for MET (45% ± 64% increase vs. 7% ± 21% decrease, *p* = 0.04; Figure 7a) but not for HEEL (165% ± 180% increase vs. 63% ± 90% increase, *p* = 0.156).

For MG for a priori planned comparisons, there were no significant differences between conditioned MEP vs. CMEP (HEEL: 102% ± 117% increase vs. 120% ± 79% increase, *p* = 0.647; MET: 16% ± 28% decrease vs. 7% ± 48% increase, *p* = 0.24; Figure 7b).

Finally, for TA, the conditioned CMEP was significantly larger than the conditioned MEP for HEEL (165% ± 135% increase vs. 2% ± 32% decrease, *p* = 0.012; Figure 7c), but not for MET (206% ± 251% increase vs. 33% ± 89% increase, *p* = 0.069).

## 4 DISCUSSION

Our findings demonstrate that foot sole cutaneous stimulation at two different functionally important locations (heel and metatarsal) modulates both cortical and spinal excitability to the plantarflexors and dorsiflexors of the ankle. Stimulation of the heel location of the foot sole significantly increased MEP amplitudes in the plantarflexor muscles, whereas stimulation to the metatarsal location resulted in no significant differences; dorsiflexor MEPs were unchanged following stimulation at either foot location. The subcortically evoked CMEPs were similarly modulated, with CMEP amplitudes to the plantarflexors larger following heel stimulation but unchanged following metatarsal stimulation. The differences between the relative MEP and CMEP changes suggests that the modulation of corticospinal excitability to the plantarflexors and dorsiflexors is likely mediated at both spinal and supraspinal levels.

### 4.1 Foot sole cutaneous stimulation and corticospinal excitability

The stimulation of cutaneous afferents has been shown to evoke a complex reflex that consists of multiple excitatory and inhibitory phases, each with their own latency (Aniss et al., 1992; Delwaide et al., 1981; Gibbs et al., 1995). The long latency response to dorsiflexors has been suggested to be mediated, at least in part, by a transcortical pathway (Nielsen et al., 1997). If true, it should be expected that the stimulation of cutaneous afferents is capable of altering corticospinal excitability, and in upper limb studies using TMS, this has been demonstrated (there is a scarcity of similar investigations in the lower limb). Across this upper limb research, many studies have shown that corticospinal excitability decreases following cutaneous afferent stimulation (Clouston et al., 1995; Inghilleri et al., 1995; Maertens de Noordhout et al., 1992; Manganotti et al., 1997; Rogić Vidaković et al., 2020; Tamburin et al., 2001), although some have shown no effect (Komori et al., 1992). Intuitively, it therefore could have been hypothesized in the present study that foot sole simulation would simply inhibit corticospinal excitability to the plantarflexors and dorsiflexors. However, upper and lower limbs have different functional roles, and in non‐TMS studies of the lower limb, the cutaneous reflex response is far more complex than widespread inhibition. Nakajima et al. (2006) demonstrated that plantarflexor (both soleus and medial gastrocnemius) muscle activity was facilitated following cutaneous stimulation of the heel but inhibited following forefoot (metatarsal) stimulation. In contrast, the tibialis anterior was facilitated by forefoot stimulation and inhibited by heel stimulation. It was suggested that this location dependent response serves a functional role, such as during different phases of the gait cycle, where during heel contact or push off, specific activation of skin regions would reflexively engage appropriate muscles (Nakajima et al., 2006; Yang & Stein, 1990). This is supported by further work by Nakajima and colleagues (Nakajima et al., 2016), where phase specific modulation of lower limb muscles was demonstrated following cutaneous stimulation over foot sole regions.

Given that muscle activity represents the net output of the motor pathway, and is sometimes even used as a surrogate measure of corticospinal function, it was hypothesized that MEP amplitudes in the present study would demonstrate a similar behaviour to the previously documented EMG reflex responses (Nakajima et al., 2006). This hypothesis was partially confirmed; both plantarflexors, SOL and MG, demonstrated increased corticospinal excitability following heel stimulation. However, the reductions in MEP amplitude following MET stimulation were not significant, and MEP amplitudes of the TA were unchanged with either stimulus location. The increased excitation of the plantarflexors following heel stimulation could function as a contributor to effective gait function, as has been previously proposed (Nakajima et al., 2006; Yang & Stein, 1990); heel stimulation could signal initial heel contact during walking and facilitate plantarflexor motoneurons to support the stance phase. However, this fails to explain why there were no changes in MEP amplitudes following MET stimulation, nor does it explain why corticospinal excitability to the TA was unchanged with either stimulus location.

One possible explanation is the influence of discrete stimulation location on the amplitude and polarity of the cutaneous reflex responses. While Nakajima and colleagues (2006) reported that stimulation of the heel resulted in excitation of plantarflexors and metatarsal stimulation resulted in inhibition, they also demonstrated a progression of reflex amplitude and polarity, based on discrete steps from distal to proximal or medial to lateral locations across the foot sole. In particular, they found that stimulation over the medial metatarsals evoked the largest reflex responses and a progressive decline in amplitude, and even reflex reversal, occurred with movement toward the lateral metatarsal. In the current study, we confirmed that participants were perceiving the stimulation under the region of interest, to ensure that we were activating mechanoreceptors underlying our target area. However, we did not specifically ask participants to identify the sensation in a medial‐lateral location, which may have led to variability in the population of mechanoreceptors that were contributing to the reflex response to metatarsal stimulation. Since our electrodes were placed over the 1st and 5th metatarsal heads, it is possible that the mechanoreceptors targeted were toward the middle or even lateral metatarsal region, which is where previous reports have shown reduced or absent reflexes (Nakajima et al., 2006). The decrease or absence of cutaneous reflex responses evoked with metatarsal stimulation would reduce the ability to see any change in MEP or CMEP in the FOOT STIM condition. Future studies examining a medial‐lateral progression of stimulation over the metatarsals, and its subsequent effects on MEP and CMEP amplitudes, would shed light on this possibility.

### 4.2 Foot sole cutaneous stimulation and spinal excitability

To discern excitability at the different levels of the pathway (cortical vs. spinal), we examined the influence of foot sole cutaneous stimulation on cervicomedullary motor evoked potentials (CMEPs). Previous research has shown that both the stretch reflex and the H‐reflex of the soleus are facilitated with cutaneous stimulation of the heel (Sayenko et al., 2007, 2009) but inhibited with cutaneous stimulation of the metatarsals (Knikou, 2007; Sayenko et al., 2009) or big and small toes (Pierrot‐Deseilligny et al., 1973). It has been suggested that the modulation of these reflexes following cutaneous stimulation could occur through connections with other afferent pathways, such as the withdrawal reflex (Sayenko et al., 2007). Regardless of the mechanism, these findings match remarkably well with the findings of Nakajima et al. (2006), whereby foot sole stimulation resulted in reflex reversals of the plantarflexors and dorsiflexors depending on stimulus location. It is not altogether surprising that these findings are so similar; stretch and H‐reflexes are strongly influenced by the excitability of motoneurons, while EMG is produced by the discharge properties of these recruited motor units. Thus, it could be expected that studies measuring spinal excitability would closely match studies measuring EMG. Indeed, given these collective muscle activity and spinal excitability findings, it was hypothesized in the present study that plantarflexor CMEPs would be facilitated with heel stimulation but inhibited with MET stimulation; the TA would demonstrate opposite behaviour. However, this was only partially confirmed. Although SOL and MG CMEP amplitudes did increase with heel stimulation, they were unaffected by MET stimulation; TA CMEPs, while increased with cutaneous stimulation, showed no location specificity.

There are several potential reasons why the present findings are not in full agreement with previous work, the first being that the aforementioned stretch reflex and H‐reflex studies were all conducted during sitting (Knikou, 2007; Pierrot‐Deseilligny et al., 1973; Sayenko et al., 2007, 2009). Similar to our MEP amplitude results, there may be task‐dependent factors that influence the effect of cutaneous stimulation on CMEP amplitudes. Second, while CMEPs and the H‐reflex are both used as measures of spinal excitability, CMEPs are believed to be not affected by the same presynaptic inhibitory inputs which can strongly influence stretch/H‐reflexes (Nielsen & Petersen, 1994; Taylor, 2006). Third, although CMEP amplitudes often do change in the same direction as changes in muscle activity, there are numerous examples of dissociation between the two measures (Collins et al., 2017; Forman et al., 2014, 2015, 2016; Lockyer et al., 2020; Spence et al., 2016). As mentioned previously, EMG is produced by the discharge properties of *recruited* motor units; CMEPs reflect the excitability of both active and inactive motor units within the motoneuron pool. As an example, it is possible that the soleus cutaneous reflex was indeed lower (reduced muscle activity compared to the no stimulation conditions) following MET stimulation in the present study (the low threshold motoneurons were inhibited), but stimulation at the MET site may not have inhibited the motoneurons that had not yet reached firing threshold. Thus, when these higher threshold motoneurons were activated by cervicomedullary electrical stimulation, no change in CMEP amplitudes was observed. This notion is supported by evidence that input from cutaneous afferents is not spread equally across the motoneuron pool. Cutaneous afferents have been shown to have differential effects on high and low threshold motoneurons (Aniss et al., 1992; Kanda et al., 1977; LaBella et al., 1989), meaning that their activity might uniquely influence experimental measures depending on what portion of the available motor pool is being investigated. It should be noted, however, that cutaneous reflexes were not in fact measured in the present study; all discussion of CMEP and EMG dissociation is made under the assumption that cutaneous reflex changes would have been similar to that in the study by Nakajima et al. (2006). Future investigations examining both EMG cutaneous reflexes and evoked potentials would be a novel advancement on this work.

### 4.3 Supraspinal mechanisms

Although corticospinal excitability was modulated in the present study, TMS alone is incapable of identifying the source of these changes. For this reason, CMEPs were also elicited to help decipher changes in corticospinal excitability as being driven by supraspinal pathways, spinal pathways, or perhaps a combination of both. For the SOL at the MET location, and the TA at both locations, the increase in CMEP amplitude following stimulation was larger than the change in the MEP amplitude (Figure 7). If spinal excitability increases despite no change in corticospinal excitability, or it increases significantly more than a modest increase in corticospinal excitability, this likely indicates that excitability from supraspinal sources decreased. In contrast, the relative changes in the MEP and CMEP amplitudes for the MG were not significantly different, which could mean that the change in spinal excitability accounts for most, if not all, of the change in corticospinal excitability.

Although evidence is growing that a transcortical pathway contributes to at least part of the cutaneous reflex, it is still unclear how cutaneous stimulation of the foot influences the corticospinal tract. Work by Nielsen et al. (1997) found that magnetic stimulation, but not electric stimulation of the motor cortex, exhibited additive facilitation of the H‐reflex following cutaneous afferent stimulation of the nerves supplying the dorsum of the foot (Nielsen et al., 1997). Transcranial electric stimulation (TES) is believed to activate the corticospinal tract fibers directly, within millimeters of the cell body, while TMS tends to activate the corticospinal neurons trans‐synaptically through more superficial cortical neurons (Day et al., 1989; Di Lazzaro et al., 1998; Edgley et al., 1990; Nielsen et al., 1995). Thus, facilitation of TMS, but not TES, suggests an increase of cortical excitability following stimulation of cutaneous afferents from the dorsum of the foot. In the present study, stimulation of the sole of the foot resulted in apparent reduction in supraspinal excitability. This could potentially be explained by changes in intracortical pathways, but literature on this topic does not seem to support it. In upper limb studies, although MEP amplitudes decrease following cutaneous stimulation (Clouston et al., 1995; Inghilleri et al., 1995; Maertens de Noordhout et al., 1992; Manganotti et al., 1997; Rogić Vidaković et al., 2020; Tamburin et al., 2001), cutaneous stimulation appears to consistently result in decreased short‐interval intracortical inhibition (SICI) and increased intracortical facilitation (ICF) (Aimonetti & Nielsen, 2001; McDonnell et al., 2007; Ridding et al., 2005; Ridding & Rothwell, 1999; Smith et al., 2011), which would seemingly oppose a net decrease in supraspinal excitability. However, as mentioned, the above studies were all performed in the distal upper limb; we are unaware of any similar investigations performed in the lower limb. Future lower limb work using paired‐pulse TMS techniques alongside preceding cutaneous stimulation would be an important next step in this area of research.

### 4.4 Functional significance

This study provides evidence that reflexes in lower leg muscles evoked by stimulation of cutaneous afferents of the foot sole are influenced by a transcortical pathway. During standing, there appears to be a withdrawal of cortical drive, which is dependent on the muscle assessed and the location of foot sole stimulation. These findings not only demonstrate the importance of cutaneous inputs in human motor control but also offer new possibilities in rehabilitation for neurological patients, as they highlight the potential for greater cortical control in tasks such as quiet standing. The motor cortex has generally been accepted to play a minor role in postural control; posture has been thought to be largely regulated by subcortical structures (Prentice & Drew, 2001). Given the evidence that a transcortical pathway likely contributes to part of the cutaneous reflex, current rehabilitation strategies for people suffering from impaired postural control may benefit from optimizing a more inclusive target for adaptations in this cutaneous reflex pathway. Such modifications to current rehabilitative guidelines could lead to improved functional outcomes, although more research is needed on this topic.

Additionally, the influence of sensory information, including that provided by cutaneous receptors, on the control (or steering) of locomotor outputs such as walking have long been thought to act primarily at the spinal cord level (Zehr et al., 2014). The results of the present study that a transcortical pathway is likely involved in the production of a cutaneous reflex suggest that cutaneous activity may have a more complex and sophisticated influence on the control of locomotor outputs. It is possible that this sensory information is integrated with other sensory sources, such as spindle activity and vision, in higher brain structures to aid in step adjustments during walking.

### 4.5 Methodological considerations

Unconditioned MEP and CMEP amplitudes (NO FOOT STIM) were significantly different in the SOL and the TA, meaning that the two measures were likely examining different portions of the available motor pool. Given that cutaneous afferents can differentially affect high and low threshold motoneurons (Aniss et al., 1992; Kanda et al., 1977; LaBella et al., 1989), the larger MEPs (Figure 3) may have been influenced differently compared to the smaller CMEPs (Figure 5) following the same cutaneous afferent stimulation. However, it should be noted that, while the unconditioned MEPs and CMEPs were statistically different, all the evoked potentials in the present study were quite small (group means <0.5 mV). It is therefore unlikely that a nonuniform effect of cutaneous afferent activity would have been a major contributing factor in the present findings.

Additionally, it should be clarified that, while the present findings may have implications for the control of locomotor outputs (the possibility of cutaneous activity influencing gait was an important rationale for conducting this investigation), this study was conducted while participants were standing statically. Given that cutaneous reflexes demonstrate certain task‐dependent behaviours (Nakajima et al., 2006), it is possible that results found during standing may not manifest similarly in other tasks, namely gait. This point should be considered while interpreting the present study's findings.

## 5 CONCLUSION

Both corticospinal and spinal excitability to the plantarflexors and dorsiflexors were modulated by cutaneous stimulation of the foot sole, an effect that was specific to the location of applied stimulation. The mechanisms behind these changes likely occurred at both a supraspinal and spinal level, and it is possible that intracortical pathways may have been involved. Not only do these findings provide additional evidence that a transcortical pathway likely contributes to part of the foot sole cutaneous reflex, but they also demonstrate a possible functional importance of cutaneous stimulation in motor control; the withdrawal of cortical drive following cutaneous afferent stimulation could allow for other pathways to better respond to cutaneous stimulation originating at the foot sole. Further research is needed to elucidate the specific mechanisms behind these processes.

## CONFLICT OF INTERESTS

The authors declare no conflict of interests.

## ETHICS STATEMENT

All experimental procedures were approved by the research ethics board at the University of Guelph (REB#16‐12‐520). All subjects provided their written consent prior to participation in the study.

## AUTHOR CONTRIBUTIONS

All experiments were conducted in Dr. Leah Bent‘s laboratory on the University of Guelph campus. GG and LRB contributed to the design of the work and to the acquisition, analysis, and interpretation for the work. DAF, JR, and JLT contributed to the analysis and interpretation of the work. All authors contributed to drafting the work and revising it critically for important intellectual content.
